# Shannon Entropy of Binary Wavelet Packet Subbands and Its Application in Bearing Fault Extraction

**DOI:** 10.3390/e20040260

**Published:** 2018-04-09

**Authors:** Shuting Wan, Xiong Zhang, Longjiang Dou

**Affiliations:** Department of Mechanical Engineering, North China Electric Power University, Baoding 071003, China

**Keywords:** bearing diagnosis, FSK, BWPT, Shannon entropy

## Abstract

The fast spectrum kurtosis (FSK) algorithm can adaptively identify and select the resonant frequency band and extract the fault feature via the envelope demodulation method. However, the FSK method has some limitations due to its susceptibility to noise and random knocks. To overcome this shortage, a new method is proposed in this paper. Firstly, we use the binary wavelet packet transform (BWPT) instead of the finite impulse response (FIR) filter bank as the frequency band segmentation method. Following this, the Shannon entropy of each frequency band is calculated. The appropriate center frequency and bandwidth are chosen for filtering by using the inverse of the Shannon entropy as the index. Finally, the envelope spectrum of the filtered signal is analyzed and the faulty feature information is obtained from the envelope spectrum. Through simulation and experimental verification, we found that Shannon entropy is—to some extent—better than kurtosis as a frequency-selective index, and that the Shannon entropy of the binary wavelet packet transform method is more accurate for fault feature extraction.

## 1. Introduction

Rolling bearings are one of the most widely used parts in mechanical equipment. Their operation condition affects the working state of the whole system. The fault identification and diagnosis of rolling bearings are of great significance in ensuring the safe and reliable operation of mechanical equipment. The separation of faulty components from strong background noise is a difficult problem in the field of mechanical fault diagnosis.

**Figure 1:**
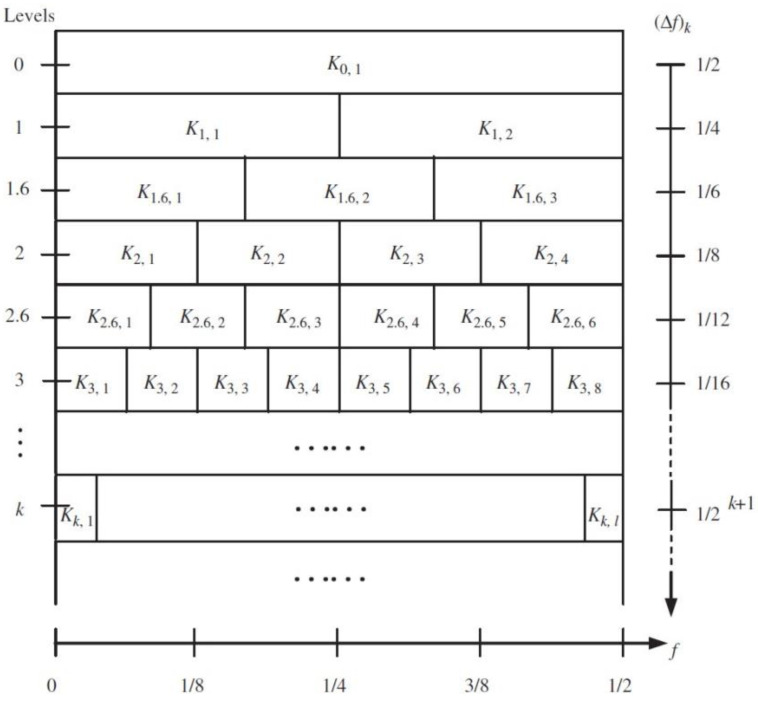
The paving of the FSK.

The concept of spectrum kurtosis (SK) was presented by Dwyer. The basic principle was to calculate the kurtosis value of each spectral line; the value of different kurtoses could respond to the size of the transient impact. SK was rarely used until Antony et al. proposed a formal definition. They also proposed an estimator based on a short-time Fourier transform. The estimator can help link theoretical concepts to practical applications [1]. After that, Anthony et al. continued to work on how the SK can be used in dealing with nonstationary signals. Additionally, the concept of the kurtogram was introduced in their paper. The optimal band-pass filters can be selected by the kurtogram as a prelude to envelope analysis [2]. Then, in order to simplify the operation process of the kurtogram and to improve the speed of the algorithm and make it meet the requirements of industrial monitoring, Antony et al. proposed a fast spectral kurtosis (FSK) algorithm, which simplifies the process of selecting the optimal band (f,Δf). The structure of the fast spectral kurtosis algorithm is as follows: the first layer of the algorithm decomposes the signal in a binary tree structure, and the second layer is decomposed into a 1/3 tree structure; the rest can be deduced by analogy [3].The paving of the fast spectrum kurtosis (FSK) is shown in Figure 1.

Since then, scholars have begun to study how to improve the kurtogram. These improvements are mainly aimed at two aspects of the algorithm. One aspect is the improvement of the frequency band segmentation method, which seeks to find an excellent method for making the selection of the center frequency and bandwidth more accurate and more adaptive. The other aspect involves improving the calculation method of the index and trying to find a method to replace the traditional kurtosis.

Wang and Liang improved the kurtogram by adapting the size of the adaptive bandwidth and center frequency. They implemented the algorithm by expanding an initial window in the frequency axis via successive attempts to merge it with its subsequent translated windows [4]. Lei et al. proposed an improved kurtogram, in which the wavelet method was used to replace the traditional short time Fourier transform (STFT) or finite impulse response (FIR) filters in the traditional kurtogram algorithm. The improvement can improve the accuracy of the selective resonance frequency band in the event of large noise [5]. Based on Lei’s improvement, Wang made a further improvement to the kurtogram. The kurtosis value was calculated based on the power spectrum of the envelope of the signals. This improvement had a good effect on the low signal-to-noise ratio and on non-Gaussian noise interference [6]. Then, Tse and Wang made a deeper study of the kurtogram. A new concept called a sparsogram was proposed, which was constructed using sparsity measurements of the power spectra from the envelopes of the signal [7]. There are still two shortcomings in this algorithm: one is that the fixed central frequency and bandwidth cannot cover the entire resonant frequency band. The other shortcoming is that the bearing resonant frequency band may be split into two adjacent imperfect orthogonal frequency bands, which reduce the bearing fault features. Considering the above, Tse and Wang made an improvement in their second paper. They presented an automatic selection process for finding the optimal complex Morlet wavelet filter with the help of a genetic algorithm that maximizes the sparsity measurement value [8]. Chen presented an improved version of FSK named a “fast spatial-spectral ensemble kurtosis kurtogram”. Discrete quasi-analytic wavelet tight frame (QAWTF) expansion methods were incorporated in the kurtogram algorithm as a detection filter instead of as an STFT or FIR filter. Moreover, an enhanced signal impulsiveness evaluating indicator, named a “spatial-spectral ensemble kurtosis (SSEK)” was used as the quantitative measure to select optimal analysis parameters [9]. Borghesani et al. presented an optimal indicator—the ratio of cyclic content (RCC)—for the choice of the selectivity in the cyclic frequency domain. Additionally, they compared the RCC with the more traditional kurtosis-based indicators [10]. Barszcz et al. propose the protugram method, which is based on the kurtosis of the envelope spectrum amplitudes of the demodulated signal, instead of the kurtosis of the time signal [11]. A new index called spectral L2/L1 was proposed by Wang. The calculation method of the index is changed by adjusting the values of *p* and *q*, which makes the index more general than spectral kurtosis [12].

Entropy is derived from the concept of thermodynamics. Entropy can reflect a direct relationship between the size of the information and its uncertainty. It is precisely because entropy can reflect the measure of probabilistic and repetitive events that it is used so widely in various fields. For instance, the concept of entropy can be used in the design process of complex mechanical systems. Villecco et al. gave the entropic measure of epistemic uncertainties, which can be used in multibody system models [13,14]. At the same time, entropy was also applied to the signal processing of rotating machinery [15,16,17,18].

Antoni then introduced entropy to the kurtogram algorithm. After comprehensively considering the periodic and impact characteristics of the signal, Antoni proposed the squared envelope (SE) infogram, squared envelope spectrum (SES) infogram, and SE_1/2_/SES_1/2_ infogram. Specifically, the SE infogram was used to calculate the negative entropy of the square envelope as an index, and the same SES infogram was used to calculate the negative entropy of the square envelope spectrum as an index [19]. Following this, scholars have done a great deal of work on the improvement of the infogram. These scholars mainly include Li, Xu, and Wang. Li et al. proposed the multiscale clustering grey infogram (MCGI), which was based on combining both negentropies in a grey fashion using multiscale clustering. First, the signal was decomposed into a multiple scale according to the Fourier spectrum, and fine segments were grouped using hierarchical clustering in each scale. Meanwhile, both time-domain and frequency-domain spectral negentropies were taken into account to guide the clustering through a grey evaluation of both negentropies [20]. Xu et al. proposed the multiscale fractional order entropy (MSFE) infogram, which can help in the selection of the optimal frequency band for extracting the repetitive transients from the whole frequency bands [21]. Wang extended the infogram to novel Bayesian inference-based optimal wavelet filtering for bearing fault feature identification [22].

In the case of strong noise interference, the FSK is often inaccurate in determining the center frequency and bandwidth of the signal resonance. In order to further improve the accuracy and efficiency of the algorithm in determining the center frequency and bandwidth, a method based on computing the Shannon entropy of wavelet subbands is proposed in this article. The new method can achieve a good control of the noise, as well as a selection of the center frequency that is more accurate and faster, benefiting from the good frequency band segmentation characteristics of the wavelet packet and the reliability of the Shannon entropy index.

## 2. The Limitation of the Kurtosis Index

The previous improvement of FSK mainly focused on the frequency band segmentation, where the ideal effect was achieved. However, there is still a problem with the kurtosis index. The limitation of the kurtosis index mainly manifests itself in two aspects. The first is that kurtosis can easily be disturbed by noise, and the second is that kurtosis is sensitive to random knocks. The deficiency of the kurtosis index is verified by a simulation signal, and the simulation signal is defined as Equation (1):

(1) $$\left\{ \begin{array}{l} y_{1}=\sum_{i=1}^{n}Ae^{-\xi {\left[t-q_{i}(t)/f_{outer}\right]}^{2}}\cdot \sin(2\pi f_{o}t)\\ y_{2}=A_{1}e^{\left[-b_{1}\times \left(t-t_{1}\right)\right]}\cdot \sin\left[2\pi f_{1}\cdot \left(t-t_{1}\right)\right]+A_{2}e^{\left[-b_{2}\times \left(t-t_{2}\right)\right]}\cdot \sin\left[2\pi f_{2}\cdot \left(t-t_{2}\right)\right]\\ y_{3}=n(t)\\ Y=y_{1}+y_{2}+y_{3} \end{array}\right.,$$

where *c* is the center of the resonance frequency band, $f_{outer}=50\;\mathrm{Hz}$ is the characteristic frequency, ξ is the damping ratio, A is the amplitude, and $q_{i}\left(t\right)=\left\lfloor t\cdot f_{i}\right\rfloor$(i=1,2,…,n). $A_{1}=3$ and $A_{2}=6$ are the amplitudes, $b_{1}=-260$, $b_{2}=-360$, $t_{1}=0.18$, $t_{2}=0.5$, $f_{1}=1000$, $f_{2}=2600$. n(t) is the Gaussian white noise. The parameters of the simulation signal are shown in Table 1.

**Figure 2:**
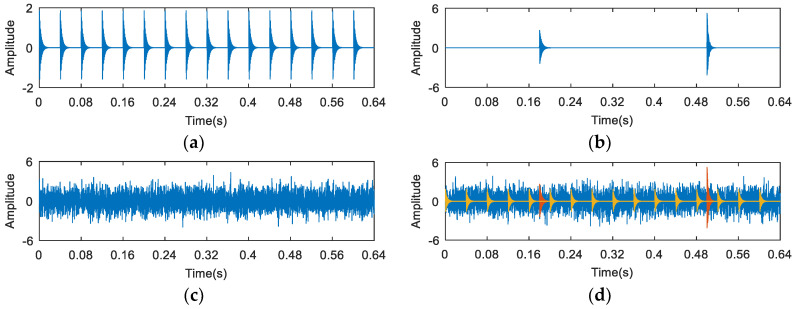
The waveforms of (**a**) fault impulses; (**b**) random knocks; (**c**) the noise signal; and (**d**) the synthetic signal.

The waveforms of the simulation signal are shown in Figure 2. The synthetic signal (Figure 2d) is composed of three parts. Figure 2a shows a periodic signal, which is used to simulate the fault impact. Figure 2b represents the random knocks, and Figure 2c shows the Gaussian noise.

**Figure 3:**
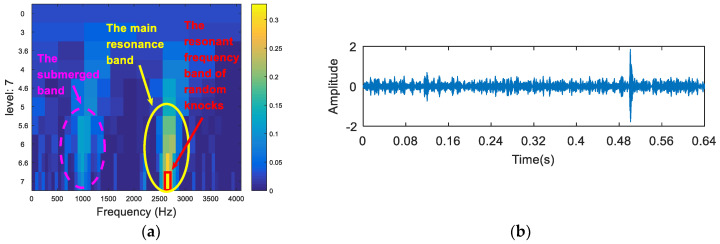

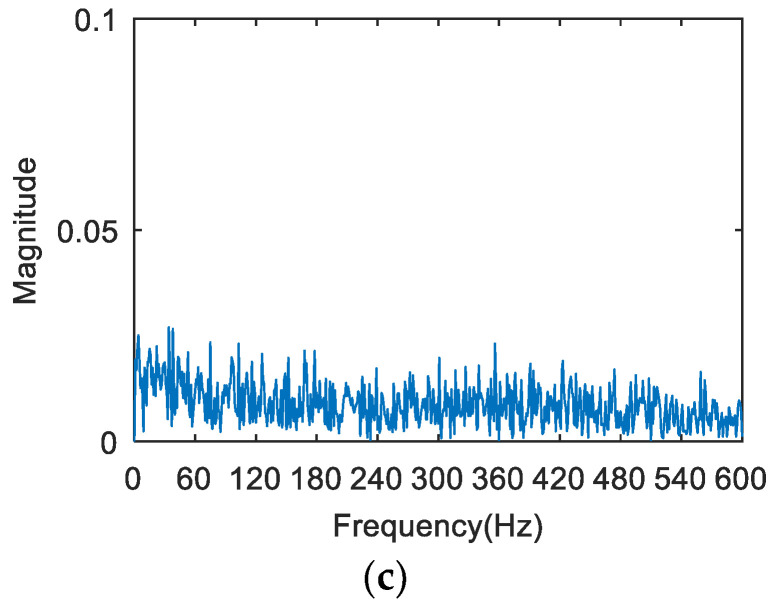
(**a**) The kurtogram of the synthetic signal; (**b**) the signal after band pass filtering; (**c**) the envelope spectrum of (**b**).

The synthetic signal was analyzed by the FSK. The results are shown in Figure 3. As can be seen from Figure 3a, the central frequency of the resonant frequency band determined by the FSK was 2650 Hz, while the actual resonant frequency band of the simulation signal was 1000 Hz. The band pass filter was carried out to obtain the resonance frequency band shown in Figure 3a, and the envelope spectrum of the filtered signal was analyzed. The results are shown in Figure 3b,c. It can be seen that the filtered signal is the random knocks component in the synthetic signal. Therefore, the FSK presented an error under the random knocks interference.

**Figure 4:**
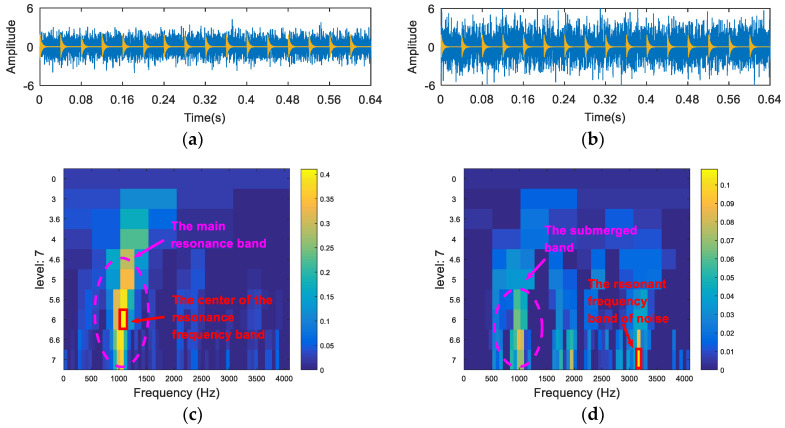
The resonance band detection using fast spectral kurtosis (FSK): (**a**) the synthetic signal with fault impulses and noise (−12 dB); (**b**) the synthetic signal with fault impulses and noise (−16 dB); (**c**) the kurtogram of (**a**); and (**d**) the kurtogram of (**b**).

Following this, the random knocks (y2) in the synthetic signal were removed. As shown in Figure 4, in order to verify the sensitivity of the kurtosis index to noise, an FSK analysis of the simulated signal was carried out under different signal-to-noise ratio (SNR) conditions by adjusting the size of the random noise. The SNR of the signal in Figure 4a was −12dB, and the SNR of the signal in Figure 4b was −16 dB. Figure 4c is the kurtogram of Figure 4a from which a resonance frequency band of 1000 Hz can be obtained. Figure 4d is the kurtogram of Figure 4b from which the resonance frequency band cannot be accurately obtained. This can be explained by the fact that the FSK method cannot extract the fault feature information under the strong noise interference due to the sensitivity of the kurtosis index to the noise.

## 3. Proposed Method

### 3.1. Binary Wavelet Packet Transform

The traditional kurtogram algorithm usually uses a fast Fourier transform (FFT) decomposition or an FIR filter as a hierarchical decomposition method. In order to improve the accuracy and efficiency of the resonance frequency band selection, more precise filters need to be incorporated into the kurtogram.

Wavelet packet transform can also be called subband tree or optimal subtree structuring. Wavelet packet analysis can provide a more precise method for signal analysis, which overcomes the problem of the wavelet analysis being inaccurate with respect to the division of high frequency bands. Moreover, it introduces the concept of basis selection based on wavelet theory. After the multilevel division of the frequency band, the best base function is selected adaptively according to the characteristics of the analyzed signal. From the viewpoint of signal processing, the WPT allows the signal to pass through a series of filters which are different in frequency but have the same bandwidth. The process can be defined by the following Equation (2):

(2) $$\left\{ \begin{array}{l} b_{i,j}=\sqrt{2}\sum_{n=0}^{K}H_{n}b_{i-1,j+n}\\ b_{i,j+l}=\sqrt{2}\sum_{n=0}^{K}G_{n}b_{i-1,j+n} \end{array}\right.,$$

where $b_{i,j}$ denotes the *j*th decomposed frequency-band signal at level *i* (*j* = 1, 2, …, *J*, where *J* is the number of the decomposed frequency-band signals and equals $2^{I}$; i=1,2,…,I, where I is the number of the decomposition levels); $H_{n}$ and $G_{n}$ are the low-pass and high-pass filters based on the wavelets, respectively.

### 3.2. Shannon Entropy

The concept of entropy was put forward by Clausius, a German physicist in 1865. It was originally used to describe the material state of “energy degradation”. It has been widely applied in thermodynamics. In 1948, Shannon extended the concept of entropy in statistical physics to the process of signal processing in order to solve the problem of quantitative measurements of information, thus creating the “information theory”. Shannon entropy can measure the disorder or uncertainty of information, and consequently it is applied to many fields. Given a random sequence $\left\{x_{1},x_{2},......,x_{n}\right\}$, the Shannon entropy is calculated according to Equation (3):

(3) $$Sh=-c\sum_{i=1}^{n}p\left(x_{i}\right)\cdot \log\left[p\left(x_{i}\right)\right],$$

where S is the Shannon entropy, $p\left(x_{i}\right)$ is the probability mass associated with the value $x_{i}$ and c is an arbitrary positive constant that dictates the units. If we allow that c equals 1/log(2), then the logarithm base is 2 and the entropy is measured in bits. It should be noted that the Shannon entropy H is non-negative and that the probabilities need to sum to 1.

**Figure 5:**
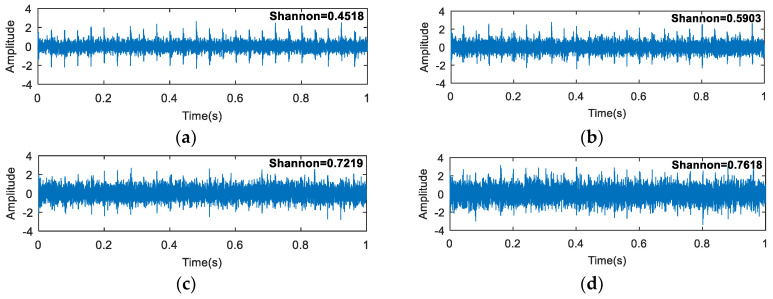

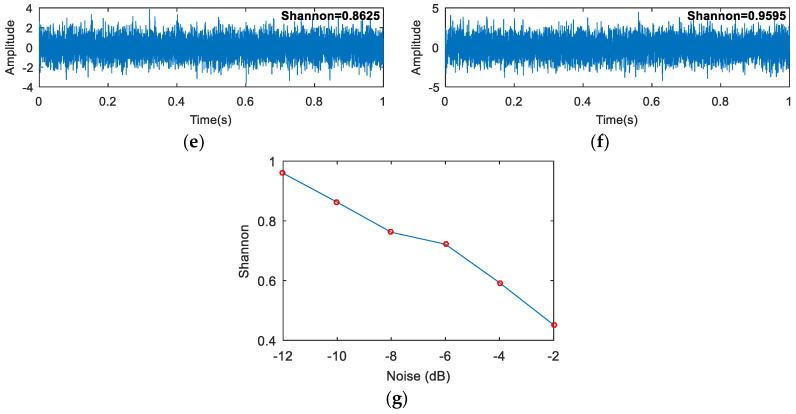
The value of the Shannon entropy under different signal-to-noise ratio (SNR): (**a**) −2 dB; (**b**) −4 dB; (**c**) −6 dB; (**d**) −8 dB; (**e**) −10 dB; (**f**) −12 dB; (**g**) the curve of the Shannon entropy with different SNR.

The different SNRs were simulated by adding varying degrees of noise to the periodic impact signal (y1). The results are shown in Figure 5. It can be seen that with the addition of noise, the value of the Shannon entropy increased; that is, with the increase of the SNR, the Shannon entropy decreased. This is because the uncertainty of the signal increases with the addition of the noise. It is fully proved that Shannon entropy can be used as an indicator to measure the periodic impact strength of the signal.

### 3.3. Process of the Proposed Method

I The theoretical characteristic frequency of the bearing fault is calculated according to the parameter of the bearing. The empirical formula is as follows:
  $$f_{i}=\frac{Z}{2}(1+\frac{d}{D}\cos\beta )\frac{N}{60}\;f_{o}=\frac{Z}{2}(1-\frac{d}{D}\cos\beta )\frac{N}{60},$$
  $$f_{\mathrm{ball}}=\frac{D}{2d}{\left(1-(\frac{d}{D}\right)}^{2}\cos^{2}\beta )\frac{N}{60}\;f_{\mathrm{cage}}=\frac{1}{2}(1-\frac{d}{D}\cos\beta )\frac{N}{60},$$
  where $f_{i}$, $f_{o}$, $f_{\mathrm{ball}}$, and $f_{\mathrm{cage}}$ respectively represent the inner ring fault, outer ring fault, rolling body element fault, and cage fault. Z is the number of the ball element, d is the diameter of the rolling element, D is the pitch diameter, N is the spindle speed, and β is the pressure angle.
II The vibration signal is decomposed by binary wavelet packet transform (BWPT), and the Shannon entropy ($Sh_{i,j}$) of each subband after BWPT is calculated. After this, the inverse of each subband Shannon entropy value is taken as Equation (4):
  (4) $$S_{i,j}=\frac{1}{Sh_{i,j}}.$$
III We then generate an entropy spectrum with the inverse value ($S_{i,j}$) of the Shannon entropy of each wavelet packet. The subband of the maximum *S* value (the minimum Shannon entropy) is selected as the optimal resonance frequency band.
IV The appropriate central frequency and bandwidth are selected to filter the resonance frequency band determined in (III). The envelope spectrum of the filtered signal is analyzed. Then, the characteristic spectral lines in the spectrum are compared with the theoretical frequency from (I), and the type of the fault is determined.

**Figure 6:**
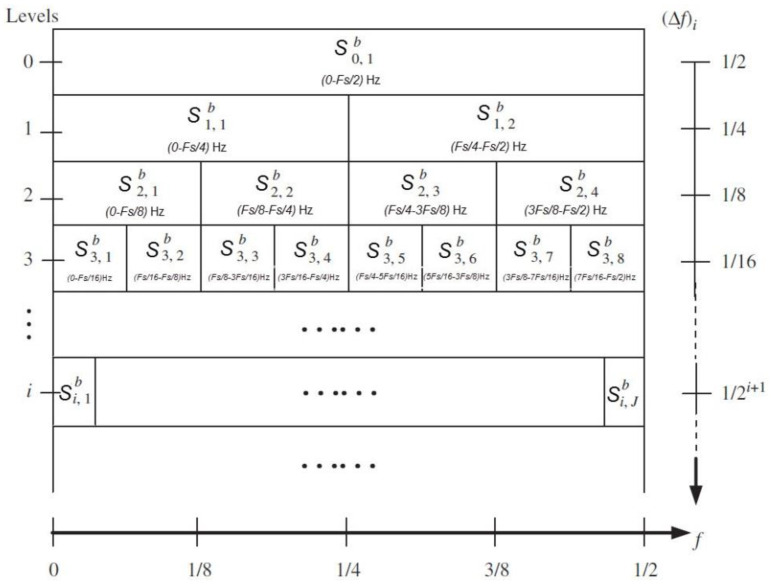
The paving of the proposed method.

The paving of the proposed method is shown in Figure 6.

## 4. Simulation Analysis

The simulation model of the inner ring fault signal is defined as Equation (5) [23]:

(5) $$\left\{ \begin{array}{l} x(t)=s(t)+n(t)=\sum_{i}A_{i}h(t-iT-\tau_{i})+n(t)\\ A_{i}=A_{0}\cos(2\pi f_{r}t+\phi_{A})+C_{A}\\ h(t)=e^{-Bt}\cos(2\pi f_{n}t+\phi_{\omega }) \end{array}\right.,$$

where $\tau_{i}$ is the minor fluctuation of the *i*th shock relative to the period *T*; $A_{i}$ is the amplitude modulation with $1/f_{r}$ as a cycle; h(t) is an exponential decay pulse; B is the attenuation coefficient of the system; $\phi_{A}$ and $\phi_{\omega }$ are the initial phases. The parameters of the inner ring simulation signal are shown in Table 2.

**Figure 7:**
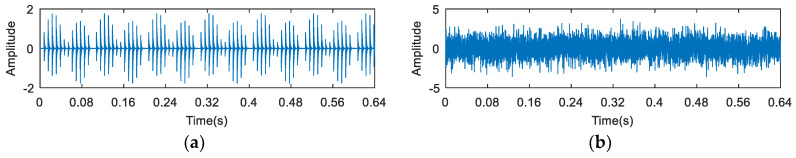

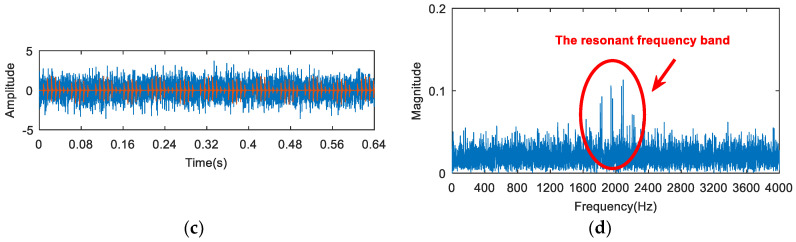
The waveforms of (**a**) the inner fault impulses; (**b**) the noise signal; (**c**) the synthetic signal; and (**d**) the spectrum of (**c**).

The waveform of the simulation signal of the inner ring is shown in Figure 7a; Figure 7b shows the Gaussian noise; Figure 7c shows the synthetic signal. Figure 7d shows the spectrum diagram of Figure 7c. It can be seen from Figure 7d that the inner impact characteristics were submerged by noise and that the natural frequency of the synthetic signal was at about 2000 Hz.

**Figure 8:**
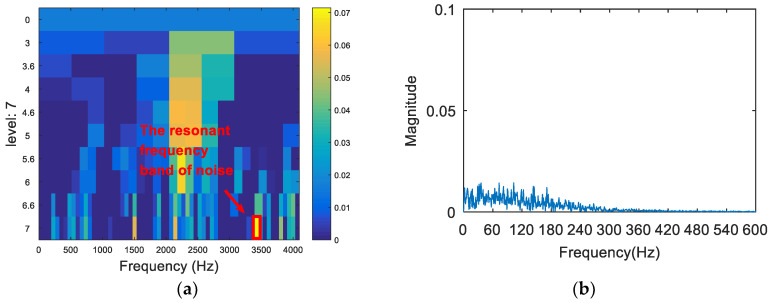
The resonance band detection using FSK: (**a**) the kurtogram, and (**b**) the envelope spectrum of the signal after band pass filtering.

**Figure 9:**
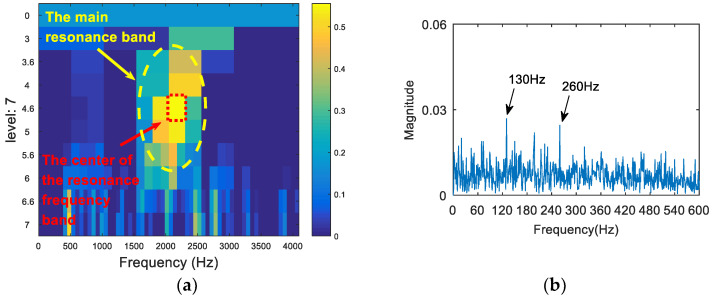
The resonance band detection using the infogram: (**a**) the SE_1/2_/SES_1/2_ infogram; and (**b**) the envelope spectrum of the signal after band pass filtering.

**Figure 10:**
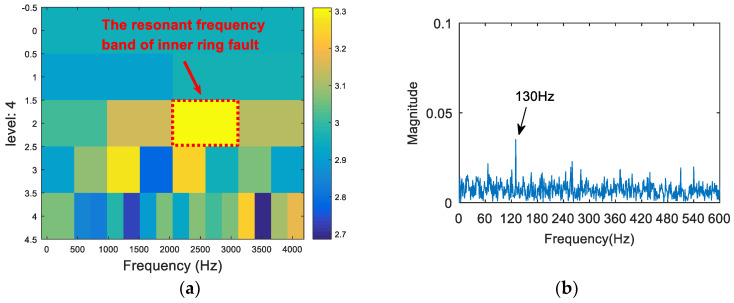
The resonance band detection using the kurtosis spectrum of the wavelet packet: (**a**) the kurtogram; and (**b**) the envelope spectrum of the signal after band pass filtering.

**Figure 11:**
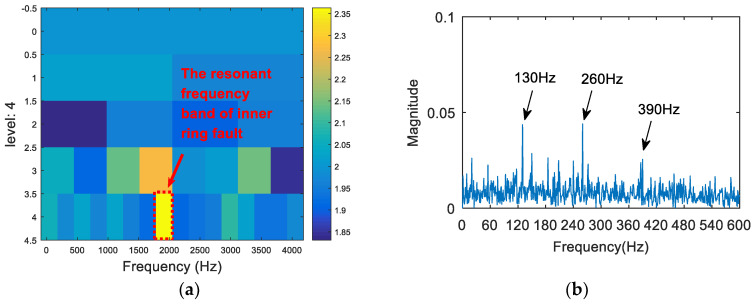
The resonance band detection using the proposed method: (**a**) the Shannon entropy spectrum of the wavelet packet; and (**b**) the envelope spectrum of the signal after band pass filtering.

The synthetic signal was analyzed by the FSK. The results are shown in Figure 8. As can be seen from (a), the central frequency of the resonant frequency band determined by the FSK was 3460 Hz, while the actual resonant frequency band of the simulation signal was 2000 Hz. The band pass filter was carried out to obtain the resonance frequency band shown in Figure 8a, and the envelope spectrum of the filtered signal was analyzed. The result is shown in Figure 8b. There was no useful information in the envelope spectrum. Therefore, the FSK method did not extract the fault feature information. Then, the synthetic signal was analyzed via the SE_1/2_/SES_1/2_ infogram, and the results are shown in Figure 9. As can be seen from Figure 9a, the central frequency of the resonant frequency band was about 2100 Hz. It can be seen from Figure 9b that the envelope spectrum of filtered signal could extract the fault characteristic frequency and the frequency doubling, but there was still large noise interference. Following this, the synthetic signal was analyzed via the kurtosis spectrum of the wavelet packet, and the results are shown in Figure 10. As can be seen from Figure 10a, the central frequency of the resonant frequency band was 2500 Hz, while the bandwidth was Fs/8. There was a certain error between the central band of the resonance and the actual value 2000 Hz, and the bandwidth was slightly larger. The envelope spectrum in Figure 10b also shows that, although the fault feature frequency 130 Hz was extracted, its frequency doubling components were not obvious due to a low signal-to-noise ratio. Finally, the proposed method was applied in order to deal with the synthetic signal. The results are shown in Figure 11. The resonance frequency band of the maximum Shannon entropy value is represented by the red line area in Figure 11a, where the frequency center was close to 2000 Hz and where the bandwidth was Fs/32. From the envelope spectrum (Figure 11b), it can be seen that the fault feature frequency and frequency doubling (130 Hz, 260 Hz, 390 Hz) were accurately extracted.

## 5. Experimental Analysis

### 5.1. Experiment 1

**Figure 12:**
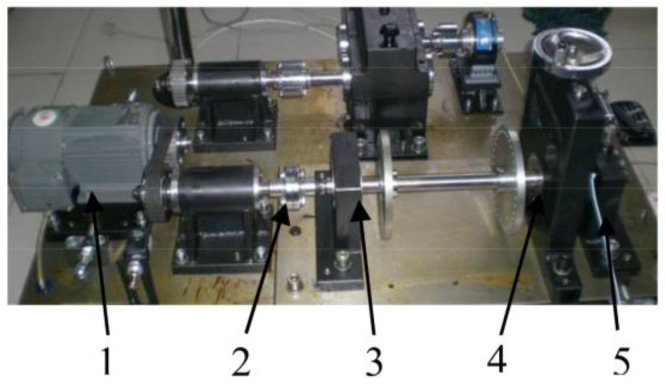
The experiment platform. 1. The drive motor; 2. The coupling; 3. The bearing housing with the normal bearing seat; 4. The loading device; 5. The bearing housing with the fail bearing seat.

**Figure 13:**
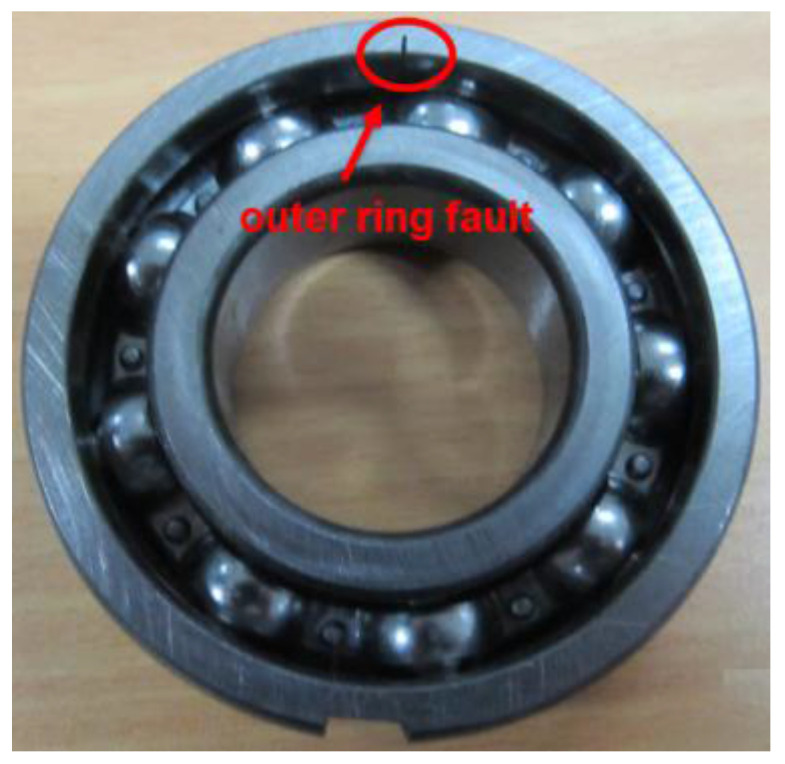
Bearing outer ring fault.

In order to further verify the effectiveness of the proposed method, the simulation experiment on the outer ring fault of the rolling bearing was completed on the simulation experiment platform. The bearing model was 6205E. The grooves, which were 1.5 mm deep and 0.2 mm wide, were processed in the outer ring in order to simulate the bearing failure. The vibration signal was collected via the acceleration sensor mounted on the bearing seat. The sampling frequency was 12,800 Hz, and the motor speed was 1466 r/min. Figure 12 shows the structural diagram of the test platform. Figure 13 shows the outer fault bearing. The bearing parameters are shown in Table 3.

The characteristic frequency of the bearing outer ring fault is calculated as:

(6) $$f_{o}=\frac{Z}{2}(1-\frac{d}{D}\cos\alpha )\frac{N}{60}=87.7\;\mathrm{Hz};$$

**Figure 14:**
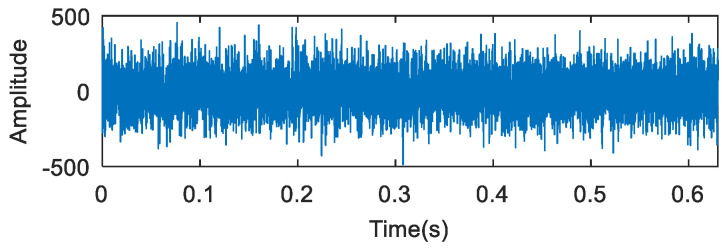
The waveforms of the outer ring fault.

8192 data points of the collected vibration signals were selected for analysis, and the time domain waveform of the outer ring fault is shown in Figure 14. 

**Figure 15:**
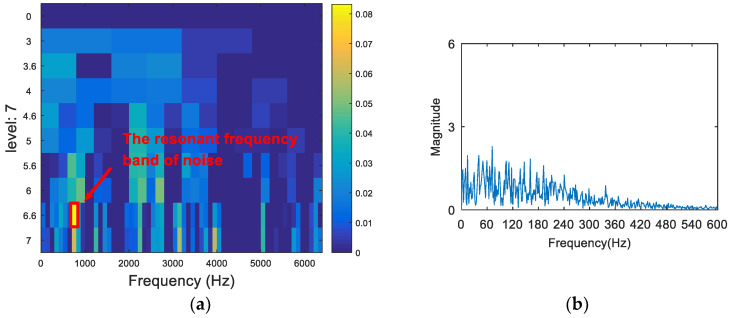
The resonance band detection using FSK: (**a**) the kurtogram; and (**b**) the envelope spectrum of the signal after band pass filtering.

**Figure 16:**
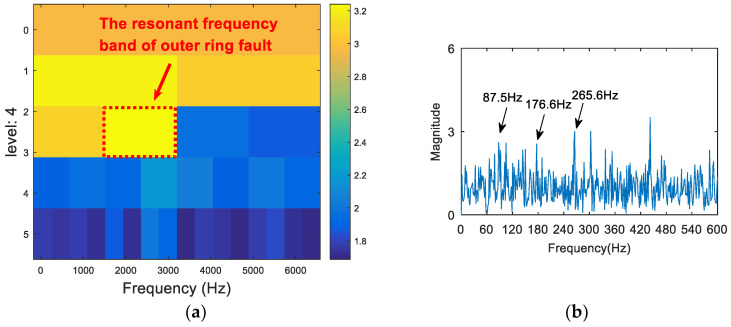
The resonance band detection using the kurtosis spectrum of the wavelet packet: (**a**) the kurtogram; and (**b**) the envelope spectrum of the signal after band pass filtering.

**Figure 17:**
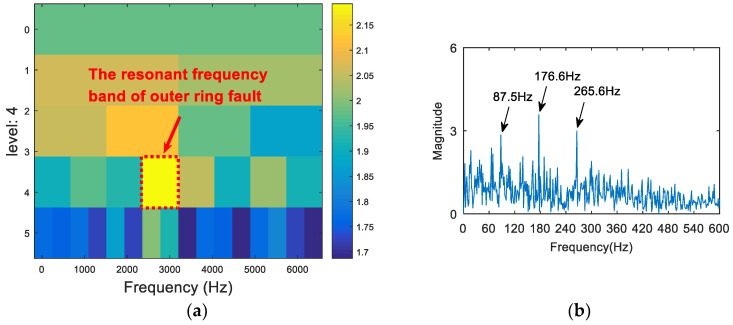
The resonance band detection using the proposed method: (**a**) the Shannon entropy spectrum of the wavelet packet; and (**b**) the envelope spectrum of the signal after band pass filtering.

The vibration signal was analyzed via the FSK. The results are shown in Figure 15. As can be seen from Figure 15a, the central frequency of the resonant frequency band determined by the FSK was 890 Hz. The band pass filter was carried out to obtain the resonance frequency band shown in Figure 15a, and the envelope spectrum of the filtered signal was analyzed. The result is shown in Figure 15b. There was no useful information in the envelope spectrum. Therefore, the FSK method did not extract the fault feature information. Following this, the vibration signal was analyzed via the kurtosis spectrum of the wavelet packet, and the results are shown in Figure 16. As can be seen from Figure 16a, the central frequency of the resonant frequency band was 2300 Hz, and the bandwidth was Fs/8. It can also be seen from the envelope spectrum in Figure 16b that the frequency and its frequency doubling components of the fault were seriously affected by the frequency of the noise, and that they were not easily discernible. Finally, the proposed method was used to deal with the vibration signal. The results are shown in Figure 17. The resonance frequency band of the maximum Shannon entropy value is shown in Figure 17a; the frequency center was close to 2750 Hz, and the bandwidth was Fs/16. From the envelope spectrum (Figure 17b), it can be seen that the fault feature frequency and frequency doubling (87.5 Hz, 176.6 Hz, 265.6 Hz) were accurately extracted.

### 5.2. Experiment 2

**Figure 18:**
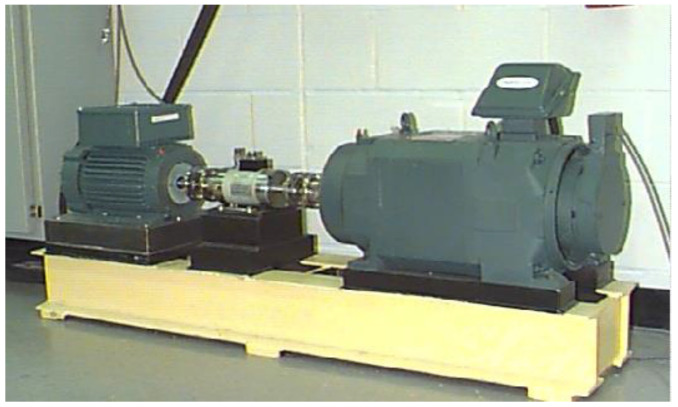
The bearing test stand at Case University.

The effectiveness of the proposed algorithm was further verified via the bearing experiment data of the rolling bearings at Case Western Reserve University in the United States [24]. The experimental equipment is shown in Figure 18. The bearing model was JEMSKF6023-2RS. The most minor dimensions of the 0.007-inch fault data were analyzed. The sampling frequency was 12 kHz, and the rotation speed of the shaft was 1772 r/min. The bearing parameter dimensions are shown in Table 4, and the frequency of each fault characteristic of the bearing is shown in Table 5.

**Figure 19:**
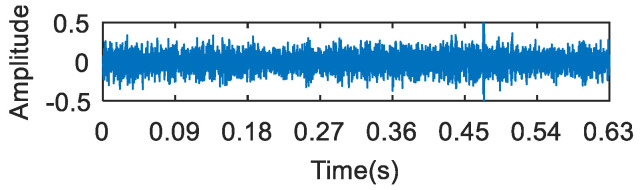
The time domain waveform of the rolling element fault.

**Figure 20:**
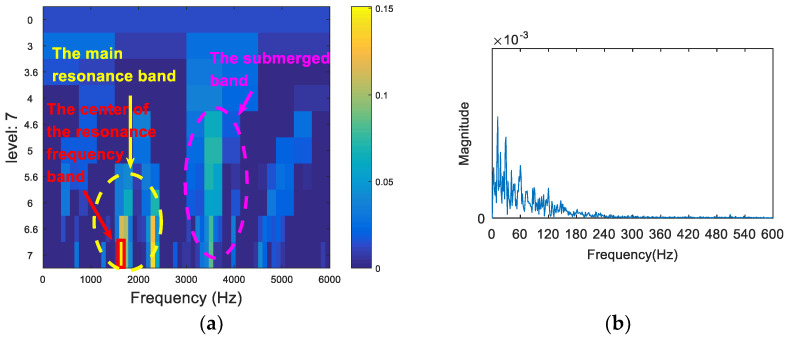
The resonance band detection using FSK: (**a**) the kurtogram; (**b**) the envelope spectrum of the signal after band pass filtering.

**Figure 21:**
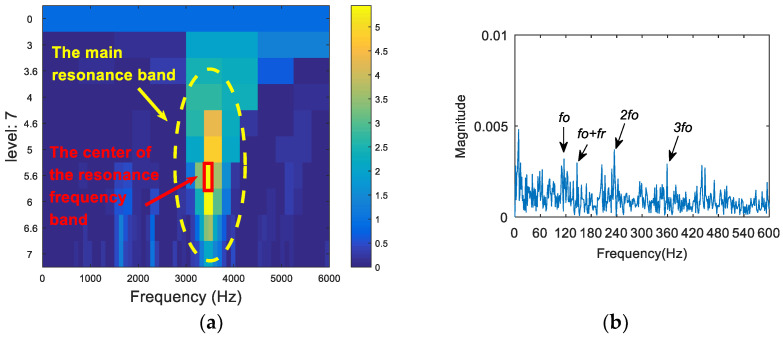
The resonance band detection using the infogram: (**a**) the SE_1/2_/SES_1/2_ infogram; and (**b**) the envelope spectrum of the signal after band pass filtering.

**Figure 22:**
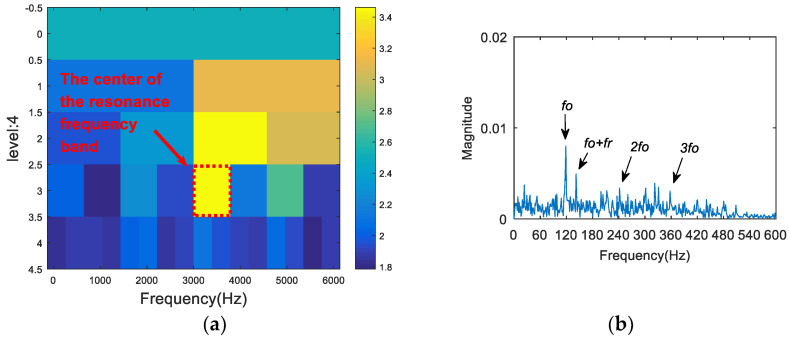
The resonance band detection using the proposed method: (**a**) the Shannon entropy spectrum of the wavelet packet; and (**b**) the envelope spectrum of the signal after band pass filtering.

The time domain waveform of the 0.007-inch rolling element fault data is shown in Figure 19. The analysis of the vibration signal by FSK is shown in Figure 20. The center frequency determined by the kurtogram method was about 1600 Hz. The selected frequency band was filtered, and the failure frequency information was not obtained from the spectrum. Following this, the vibration signal was analyzed via both the infogram (SE_1/2_/SES_1/2_) and the proposed algorithm. As can be seen from Figure 21a and Figure 22a, the two methods could both find the main resonance frequency bands. Additionally, it can be seen from the envelope spectrum (Figure 21b and Figure 22b) that the frequency information of the fault feature could be found via the two methods. Compared with the analysis results obtained through the two methods, it can be seen that the frequency doubling component (2*f*_o_, 3*f*_o_) in Figure 21b was more obvious, but that the noise interference was larger. In Figure 22b, the noise control was better and the frequency of the fault was clearer. However, the frequency doubling component was not clear because of the frequency division of the wavelet packet.

By comparing the proposed method with the infogram, we found that the proposed method has two advantages. One of them is that the wavelet packet decomposition has better spectral segmentation properties than Fourier decomposition (or FIR filter), so the envelope spectrum of this method has higher SNR. On the other hand, by calculating the Shannon entropy of wavelet packet subband instead of calculating the negentropy of the squared envelope (SE) and the squared envelope spectrum (SES), the calculation method in this paper is more direct and efficient, which can avoid envelope error generated by envelope process, and save operation time.

## 6. Conclusions

Fast spectral kurtosis is one of the most classical methods in bearing fault diagnosis. However, because of the limitations of its own algorithm, it is not possible to accurately extract fault information in the presence of strong background noise and random knocks. In view of the limitation of FSK, we proposed a new method of spectral analysis. The signal is segmented with a certain central frequency and bandwidth by using the binary wavelet packet transform, which has excellent frequency segmentation characteristics. After this, the Shannon entropy (reciprocal) of each subband is calculated. The appropriate center frequency and bandwidth have been chosen for filtering, the envelope spectrum of the filtered signal is analyzed, and the faulty feature information is obtained via the envelope spectrum. Through simulation and experimental verification, the following conclusions were obtained. Shannon entropy is—to some extent—better than kurtosis as a frequency selective index, and the Shannon entropy of the binary wavelet packet transform method is more accurate for fault feature extraction.

## Figures and Tables

**Table 1:** The parameters of the simulation signal.

Parameter Type	Parameter Values
Amplitude A	2
Rotating frequency $f_{r}$ (Hz)	20
Natural frequency (Hz)	1000
Sampling frequency (Hz)	8192
Sampling points (Hz)	8192
Fault frequency (Hz)	50

**Table 2:** The parameters of the inner fault simulation.

Parameter Type	Parameter Values
Amplitude A	1
Rotating frequency $f_{r}$ (Hz)	20
Natural frequency (Hz)	2000
Sampling frequency (Hz)	8192
Sampling points (Hz)	8192
Fault frequency (Hz)	130

**Table 3:** The parameters of the rolling bearing.

Parameter Type	Parameter Values
Inside Diameter (mm)	25
Outside Diameter (mm)	52
Ball Diameter (mm)	7.9
Pitch Diameter (mm)	39
Number of balls	9
Contact angle (°)	0

**Table 4:** Parameters of the rolling bearing.

Parameter Type	Parameter Values
Inside Diameter (mm)	17
Outside Diameter (mm)	40
Ball Diameter (mm)	6.7
Pitch Diameter (mm)	28.5
Number of balls	8
Contact angle (°)	0

**Table 5:** Fault feature frequency of the rolling bearing.

Types of Failures	Outer Ring	Inner Ring	Rolling Element
Defect frequencies (Hz)	90	145	118
